# Impact of BMI and BMI change on future drug expenditures in adults: results from the MONICA/KORA cohort study

**DOI:** 10.1186/1472-6963-13-424

**Published:** 2013-10-19

**Authors:** Christina M Teuner, Petra Menn, Margit Heier, Rolf Holle, Jürgen John, Silke B Wolfenstetter

**Affiliations:** 1 Helmholtz Zentrum München – German Research Center for Environmental Health, Institute of Health Economics and Health Care Management, Ingolstädter Landstraße 1, 85764, Neuherberg, Germany; 2 Munich School of Management – Institute of Health Economics and Health Care Management and Munich Center of Health Sciences, Ludwig-Maximilians-Universität München, Ludwigstr. 28 RG, 80539, Munich, Germany; 3 Helmholtz Zentrum München – German Research Center for Environmental Health, Institute of Epidemiology II, Ingolstädter Landstraße 1, 85764, Neuherberg, Germany

**Keywords:** Obesity, Overweight, Expenditures, Pharmaceuticals, Weight gain

## Abstract

**Background:**

The evidence on the long-term economic effects of obesity is still scarce. This study aims to analyse the impact of body mass index (BMI) and BMI-change on future pharmaceutical utilisation and expenditures.

**Methods:**

Based on data from 2,946 participants in a German population-based health survey (MONICA/KORA, 1994/95) and the follow-up study (2004/05), drug intake and expenditures were estimated using a bottom-up approach. Using univariate and multivariate methods, we analysed the impact of baseline BMI and BMI-change on drug utilisation and expenditures after 10 years.

**Results:**

The use of pharmaceuticals was more likely in moderately and severely obese compared to the normal weight group (OR 1.8 and 4.0, respectively). In those who reported pharmaceutical intake, expenditures were about 40% higher for the obese groups. A 1-point BMI-gain in 10 years was, on average, associated with almost 6% higher expenditures compared to a constant BMI.

**Conclusion:**

The results suggest that obesity as well as BMI-gain are strong predictors of future drug utilisation and associated expenditures in adults, and thus highlight the necessity of timely and effective intervention and prevention programmes. This study complements the existing literature and provides important information on the relevance of obesity as a health problem.

## Background

The global prevalence of overweight and obesity has increased significantly over recent decades. According to the World Health Organization (WHO), 1.5 billion adults were overweight in 2008. Of these, over 200 million men and nearly 300 million women were obese [1]. The results of the German Telephone Health Survey 2003 show that, in Germany, about 70% of men and 50% of women are overweight or obese. Compared with the results of the German National Health Interview and Examination Survey 1998, this implies a considerable increase in adult prevalence rates [2].

Obesity is a major public health concern, presenting a huge burden of directly and indirectly obesity-related diseases [3] as well as economic implications for society. Besides lower skill attainment [4] and worse labour market outcomes [5], these economic consequences also include higher health service expenditures – already visible in childhood [6-8] and later on in adulthood [5].

The obesity-related drug prescription costs for Medicare beneficiaries were analysed by Stuart et al. for the USA. They concluded that overweight and obese people have significantly higher drug expenditure compared with normal weight people, mainly owing to chronic diseases [9]. Two other US studies confirm these findings, reporting an increase in average prescription drug expenditures by 80.4% compared with normal weight insurants [10] and 95% greater prescription drug expenditures for morbidly obese compared with normal weight adults [11]. A further cross-sectional study in the UK showed a considerable increase in annual prescription drug expenditures with each unit increase in body mass index (BMI), which was greater in men [12]. The German study by von Lengerke et al. based on cross-sectional data also found higher expenditures for prescription drugs for severely obese compared with normal weight people [13].

However, most cost-of-illness or excess-cost studies reporting drug expenditures focused on prescription drugs, excluding patients’ out-of-pocket expenditures. Furthermore, they are mostly based on cross-sectional data and may therefore only draw conclusions regarding short-term associations of BMI and expenditures. Yet, obesity is also related to increased risk factors for several diseases that might occur with a significant time lag [14,15]. Information on the long-term influence of elevated BMI on health care expenditures and the role of weight development or maintenance is still scarce. The studies by Højgaard and colleagues examined future health care costs in relation to waist circumference and BMI in a Danish prospective cohort study. Increased waist circumference was associated with higher future health care costs [16] even for constant levels of BMI [17], but results were not reported separately for the included cost components such as drug expenditures. Dilla et al. found that medication costs for the treatment of type 2 diabetes and related comorbidities were significantly higher in Spanish type 2 diabetes patients who gained BMI compared to those without a BMI increase [18]. Based on self-reported height and weight, Thompson et al. showed for a cohort of Kaiser Permanente members aged 25–64 years that future healthcare costs were higher for overweight, but especially for obese persons. This association was specifically clear for pharmacy costs, particularly diabetes and cardiovascular medications [19].

Two German studies examined the subject of BMI and BMI change and future healthcare costs based on different surveys from the prospective MONICA/KORA cohort studies. One found higher future physician costs as well as indirect costs owing to production losses for obese participants compared with the normal weight group [20]. The second study reports that those participants who maintained overweight, gained weight or lost weight had higher outpatient physician costs compared to participants who maintained normal weight over 7 to 10 years, especially after baseline obesity [21]. However, to our knowledge, there are no publications analysing the association of BMI/BMI change and costs for pharmaceuticals for Germany so far.

To complement the existing literature, the present study aims to analyse the impact of baseline BMI and BMI change on future drug utilisation and expenditures after 10 years in German adults based on the MONICA/KORA surveys S3 and F3. This includes prescription drug expenditures as well as out-of-pocket payments.

## Methods

### Study population and sampling

The MONICA/KORA (Cooperative Health Research in the Region of Augsburg) Survey S3 conducted from October 1994 to July 1995 is a population-based health survey. Participants were randomly selected from all registered citizens of German nationality aged between 25 and 75 years in the region of Augsburg and its two surrounding counties in southern Germany. The KORA Follow-up study F3 was conducted in 2004/05, in which all S3 participants who had not died, moved abroad or to an unknown location or refused to be contacted were contacted again. Both studies were approved by the responsible ethics committee (Bavarian Medical Association, Munich).

In the S3 Survey, a sample of 4,856 participants was examined (baseline response rate: 74.9%). Of these, 3,006 individuals (61.9%) also participated in the Follow-up study F3 from October 2004 to May 2005: People were considered ineligible for F3 if they had died in the meantime (n = 405, 8%), lived too far outside the study region or were completely lost to follow-up (n = 222, 5%) or had demanded deletion of their address data (n = 270, 6%). Of the remaining 3,959 eligible people, 161 (4.1%) could not be contacted, 295 (7.5%) were unable to come because of illness or lack of time, and 497 (12.6%) were not willing to participate in this follow-up, giving a response rate of 76%. For the following analyses, all those with missing information on BMI in S3 or F3 and those with missing information on drug utilisation in F3 were excluded (n = 44). Furthermore, individuals with a BMI smaller than 18.5 kg/m^2^ were not included (n = 16) because of the small sample size. Probable underweight health conditions or even severe illness might cause problems when including those participants in the normal weight group. In sum, a complete dataset including drug utilisation and BMI was available for 2,946 (61% of the S3/baseline sample) individuals in both surveys.

### Obesity

In both studies (Baseline S3 and Follow-up F3), body weight and height of the participants were measured anthropometrically in a standard medical examination performed by trained medical staff. Calibration of measuring instruments was censured by regular inspection using standard weights. BMI was calculated for each participant as weight in kilograms/(height in metres)^2^. Following the WHO definitions, the participants were classified into four groups according to their BMI at baseline: normal weight (18.5 ≤ BMI < 25); overweight (25 ≤ BMI < 30); moderate obesity (obesity class 1: 30 ≤ BMI < 35); severe obesity (obesity class 2–3: BMI ≥ 35) [22]. BMI change was defined as the absolute BMI difference between 2004/05 and 1994/95. Those participants who changed to the underweight group (BMI < 18.5) during the 10 years (n = 6 formerly normal weight persons) were included in the normal weight group at follow-up.

### Sociodemographic and socioeconomic factors

In this analysis, gender, age and socioeconomic status in the Follow-up study (F3) were used as potential confounders. Information on gender and age was available for all participants. Determinants of socioeconomic status (SES) were assessed in a structured face-to-face health interview performed by trained medical staff [23].

SES was defined using the index compiled by Helmert and colleagues, which is recommended for the German population [24-26]. This index is based on scores for educational level, occupational status and income in the Follow-up study (F3). School education or vocational training was used for the educational level. School education was based on five categories (no school leaving certificate, primary, secondary, tertiary school or general qualification for university education), and seven categories were differentiated for vocational training ranging from no vocational training to university degree. Occupational status was grouped in a social class hierarchy proposed by Helmert and Shea [24] for the German labour market. For the equivalent household income, we used the following groups: <50%, 50–69%, 70–89%, 90–109%, 110–129%, 130–149%, 150–169%, 170–189% and >190% of the median income. Scores ranged from 1 to 9 (income, occupation) and 0 to 9 (education), respectively, and were added up to build a global score for SES. In the following analyses, participants were grouped into five SES categories [24,27,28]: lower social class = scores from 2 to 8; lower middle social class = scores from 9 to 11; middle social class = scores from 12 to 14; upper middle social class = scores from 15 to 18; and upper social class = scores from 19 to 27. To prevent loss of data that might cause systematic bias, missing values for SES (0.5%) were imputed using the Markov chain Monte Carlo function method from the SAS procedure PROC MI based on the variables educational level, occupational status and income.

### Measurement and assessment of drug utilisation and expenditures

In the F3 Follow-up study, data on the participants’ utilisation of pharmaceuticals during the previous 7 days were collected in a standardised computer-assisted personal interview [29]. For the assessment of drug utilisation and related expenditures, a rather narrow definition of pharmaceuticals was applied with reference to §2 German Pharmaceuticals Act (AMG), and ‘non-pharmaceuticals’ were excluded based on ATC (anatomic therapeutic chemical classification) groups. Specifically, vitamins and dietary supplements (ATC A11/A12) were excluded as well as ATC groups V02–V60 (varia), homoeopathic and herbal medicines. Utilisation was defined as the number of pharmaceuticals taken within the last 7 days.

Pharmaceutical expenditures were estimated based on information on the name, pharmaceutical identification number and dosage of drug intake. The pharmaceutical identification number enables a well-defined attribution of a pharmaceutical product including for example name, package size, defined daily dose (DDD) and price. First, the pharmaceutical identification number was used to derive the package price. As suggested by costing guidelines [30,31], the pharmaceuticals were priced using 2005 prices according to the national price list (available by WidO – Scientific Institute of AOK). If a definite identification of the pharmaceutical was impossible owing to missing data and the information on the drug name was imprecise (e.g. ‘pain killer’), the price of the most frequently mentioned pharmaceutical in the particular ATC group was used for the largest freely disposable package (N3) in a conservative approach. If only the agent could be identified (e.g. ‘acetylsalicylic acid/ASA’), the cheapest product in the particular ATC group was assumed.

Drug expenditures per week were calculated as follows: if pharmaceuticals were taken regularly (84%), the daily dose was multiplied by the number of days of drug intake per week (both self-reported) and then divided by the number of units per package. If pharmaceuticals were not taken regularly, the information on daily dose and days of intake were not given. In this case, we assumed one intake per week in a conservative base analysis and used the DDD. In both cases, this gives the proportion of the package that was used per week. This proportion was then multiplied by the package price, resulting in expenditures per week.

To test the sensitivity of utilisation and expenditure estimates to changes in the underlying assumptions, univariate sensitivity analyses were performed. First, to improve comparability with other studies, we also calculated the expenditures for prescription-only drugs by BMI groups. Second, in case the pharmaceuticals were not taken regularly, we assumed daily intake instead of one intake per week to show up the upper limit of estimated expenditures. Furthermore, we assessed the impact of mandatory manufacturer and pharmacy discounts for statutory health insurance on the results: first, a 6% reduction in the manufacturer’s selling price (SGB V §130a) and, second, a reduction in the pharmacy selling price of € 2 for prescription-only drugs and 5% for other drugs (SGB V §130).

In order to improve comparability with other studies, mean expenditures per week were also extrapolated to 1 year by multiplication by a factor of 52.

### Statistical analysis

In univariate analyses, the proportion of participants with pharmaceutical intake, in total and separated by ATC groups, was compared between BMI groups, and chi-square tests were conducted to assess the significance of differences. Analyses by ATC groups were performed for those ATC groups that constitute more than 5% of all pharmaceuticals in our sample (A: Alimentary tract and metabolism; B: Blood and blood-forming organs; C: Cardiovascular system; G: Genitourinary system and sex hormones; H: Systemic hormonal preparations, excluding sex hormones and insulins; M: Musculoskeletal system; N: Nervous system; R: Respiratory system).

The proportion of prescription drugs was analysed with regard to the four different BMI groups. Furthermore, the number of pharmaceuticals per person was compared between BMI groups and tested for significance using Kruskal–Wallis tests.

In order to account for non-normality of the cost data, confidence intervals (CIs) were estimated for each BMI group applying a non-parametric bootstrap approach (1,000 replications) using a percentile method.

Expenditures were shown to have a skewed distribution: whereas some participants (32%) had zero expenditures, there was a small number of people with high drug expenditures, which is typically observed for health care cost data [32,33]. To account for this skewness of the data, two-part regression models were applied.

All regression analyses were adjusted for age, sex and SES. Additionally, age was entered as a quadratic term to account for a possible non-linear effect of age. ‘Normal weight’ was the reference group for the BMI classes shown, ‘male’ for sex and ‘lower social class’ for SES.

A logistic regression model (GENMOD procedure in SAS) for the binary response variable was calculated in the first step of the two-part model predicting the odds ratio of positive expenditures in the respective obesity class. In the second step, positive expenditures were estimated using a generalised linear model (GENMOD procedure in SAS) assuming a gamma distribution with log-link function [34], which was supported by the modified Hosmer-Lemeshow Test (p = 0.46) and the Pregibon Link Test (p = 0.12). We report the exponents of regression estimates that can be interpreted as factors. We also included BMI as continuous non-linear effect in a generalised additive model, but this did not improve our model.

A separate two-part model was used to explore the relationship between future drug expenditures in the years 2004/05 and the change in BMI between 1994/95 and 2004/05 while adjusting for baseline BMI. According to the Akaike Information Criterion (AIC) the fit of this model was slightly better compared to the model without BMI change. The additional inclusion of interaction terms between baseline BMI group and BMI change was tested, but did not show an improvement, nor did the assumption of non-linearity of BMI change in a varying coefficient model.

To improve the understanding of expenditure differences, the contribution of single ATC groups to total expenditures was analysed and displayed by BMI group. Mean expenditures were adjusted for age, sex and SES using recycled predictions [35].

All statistical analyses were performed using SAS software (SAS Institute Inc., Cary, NC, USA: Version 9.2). The statistical significance level was p < 0.05.

## Results

### Sample description

Table 1 shows the characteristics of the resulting study population (n = 2,946) by BMI classes for the year 1994/95. In total, 51% of the participants were female, with significant differences between the four BMI groups. The mean age was 57.6 years, increasing with higher BMI groups. The SES status was higher among the normal weight participants compared with the overall sample. The proportion of lower SES classes is higher in higher BMI groups. The average BMI gain decreases with increasing baseline BMI, whereas standard deviations increase.

**Table 1:** Sample description at the follow-up F3 by BMI classes of the Baseline KORA survey S3 (1994/95)

		**Baseline: S3 (1994/95)**										
**Follow-up Sample**		**Overall**		**Normal weight**		**Overweight**		**Moderate obesity**		**Severe obesity**		**p-value**
		** n**	** (%)**^**b**^	** n**	** (%)**^**b**^	** n**	** (%)**^**b**^	** n**	** (%)**^**b**^	** n**	** (%)**^**b**^	
**F3 (2004/05)**		2,946	100%	1,064	35.9%	1,327	44.8%	434	14.7%	121	4.1%	
**Sex**	Men	1,444	49.0%	368	34.6%	812	61.2%	224	51.6%	40	33.1%	<.0001
	Women	1,502	51.0%	696	65.4%	515	38.8%	210	48.4%	81	66.9%	
**Socio-economic status**	Lower class	463	15.7%	106	10.0%	218	16.4%	96	22.1%	43	35.5%	<.0001
	Lower middle class	601	20.4%	191	18.0%	258	19.4%	125	28.8%	27	22.3%	
	Middle class	627	21.3%	247	23.2%	286	21.6%	74	17.1%	20	16.5%	
	Upper middle class	728	24.7%	305	28.7%	306	23.1%	91	21.0%	26	21.5%	
	Upper class	527	17.9%	215	20.2%	259	19.5%	48	11.1%	5	4.1%	
	Normal weight or underweight^a^	854	29.0%	737	69.3%	115	8.7%	2	0.5%	0	0%	<.0001
**BMI group**	Overweight	1,298	44.1%	316	29.7%	918	69.2%	63	14.5%	1	0.8%	
	Moderate obesity	599	20.3%	11	1.0%	284	21.4%	280	64.5%	24	19.8%	
	Severe obesity	195	6.6%	0	0%	10	0.8%	89	20.5%	96	79.3%	
**Age (years)**	Mean (SD)	57.6 (12.8)		52.4 (12.1)		59.9 (12.4)		62.0 (12.0)		62.0 (11.2)		<.0001
**BMI change**	Mean (SD)	0.97 (2.1)		1.14 (1.77)		0.88 (2.04)		0.98 (2.57)		0.45 (3.21)		0.0008

Underweight: BMI < 18.5; normal weight: 18.5 ≤ BMI < 25; overweight: 25 ≤ BMI < 30; moderate obesity (obesity class 1): 30 ≤ BMI < 35; severe obesity (obesity class 2–3): BMI ≥ 35.

^a^Patients who changed to the underweight group during the 10 years (N = 6 formerly normal weight persons) were included in the normal weight group at follow-up (F3).

^b^Column percentages per gender/SES are shown.

Table 1 also shows the number of patients who stay in the same obesity class from baseline to follow-up and those who change from one category to another by gaining or losing weight. Most participants remained in the normal weight or overweight category after 10 years, 31% of the normal weight participants switched to the overweight or obese classes, and 22% of the overweight participants changed to a higher BMI group status. On the other hand, over 20% of the severely obese and 15% of the moderately obese participants managed to lose weight.

### Effect of obesity on future drug utilisation and expenditures

#### Univariate analysis

In our sample, in total 6.309 drugs were reported. Table 2 displays the unadjusted results for the mean number of pharmaceuticals and the associated expenditures by BMI group. These univariate analyses show that both utilisation and expenditures increase significantly with increasing BMI. The results of sensitivity analyses show that mean expenditures are 12% lower if only prescription drugs are regarded and 4% lower if pharmacy price discounts are included. Assuming daily intake instead of one intake per week for those participants who stated irregular drug intake leads to an increase in expenditures of 29%. However, the differences between BMI classes are still significant.

**Table 2:** Drug utilisation and expenditures by baseline BMI group (unadjusted)

		**Total**	**Normal weight**	**Overweight**	**Moderate obesity**	**Severe obesity**	**p-value**^a^
	** n**	2,946	1,064	1,327	434	121	
**Number of drugs**	**Mean**	2.14	1.42	2.23	3.09	4.12	<.0001
	**SD**	2.44	1.80	2.49	2.75	3.23	
**Costs/week (base)**	**Mean**	9.48	6.94	9.69	13.63	14.51	<.0001
	**95% CI**^**b**^	[8.67-10.35]	[5.63–8.57]	[8.62–10.85]	[10.99–16.76]	[11.65–17.78]	
**Costs/week (prescription drugs)**^**c**^	**Mean**	8.35	5.67	8.58	12.80	13.55	<.0001
	**95% CI**^**b**^	[7.60-9.11]	[4.51–7.11]	[7.67–9.63]	[10.23–15.78]	[10.84–16.56]	
**Costs/week (7/week)**^**d**^	**Mean**	12.25	9.67	12.24	17.29	17.09	<.0001
	**95% CI**^**b**^	[11.34-13.20]	[8.09–11.64]	[11.03–13.52]	[14.36–20.71]	[13.96–20.61]	
**Costs/week (discounts)**^**e**^	**Mean**	9.08	6.60	9.31	13.13	13.93	<.0001
	**95% CI**^**b**^	[8.30-9.93]	[5.35–8.12]	[8.29–10.43]	[10.55–16.15]	[11.26–16.92]	
**Costs per year (base)**	**Mean**	493	361	504	709	755	<.0001
	**95% CI**^**b**^	[451–538]	[293–446]	[448–564]	[546–872]	[607–925]	

^**a**^p-values were based on Kruskal–Wallis tests.

^**b**^ Confidence intervals (CIs) were estimated based on 1,000 bootstrap replications.

^**c**^Only prescription drugs.

^**d**^In case the pharmaceuticals were not taken regularly, we assumed 7 days of intake instead of 1 (base analysis) to show up the upper limit of estimated costs as sensitivity analysis.

^**e**^Pharmacy discounts, as regularised in the Social Security Code (SGB) V §130 and §130a, were incorporated as sensitivity analysis.

68% of the participants stated drug intake during the previous week. This percentage increases significantly with increasing BMI. Detailed analyses for the most frequently taken ATC groups show that this increase is significant for the ATC groups A, C, H and M (see Table 3).

**Table 3:** Percentage of participants with drug intake by baseline BMI group

		**Total**	**By BMI group**				
			**Normal weight**	**Overweight**	**Moderate obesity**	**Severe obesity**	**p-value**^**a**^
		n = 2,946	n = 1,064	n = 1,327	n = 434	n = 121	
**Percentage**		67.9%	60.3%	68.6%	78.8%	90.1%	<.0001
**Percentage by ATC group**	A: Alimentary tract and metabolism	16.3%	8.6%	17.3%	25.1%	42.1%	<.0001
	B: Blood and blood-forming organs	13.5%	7.9%	15.7%	18.7%	21.5%	0.3732
	C: Cardiovascular system	36.9%	19.1%	40.5%	60.1%	70.2%	<.0001
	G: Genitourinary system and sex hormones	12.8%	16.7%	10.8%	9.2%	14.0%	0.2158
	H: Systemic hormonal preparations, excl. sex hormones + insulins	14.8%	12.9%	13.6%	19.8%	28.1%	0.0004
	M: Musculoskeletal system	16.5%	11.6%	16.3%	27.0%	24.8%	<.0001
	N: Nervous system	16.4%	14.9%	16.2%	20.0%	19.0%	0.5355
	R: Respiratory system	8.2%	7.7%	8.1%	9.4%	9.1%	0.4434

^a^p-values were adjusted for age, sex and SES.

#### Multivariate analysis

Table 4 displays the results of the two-step regression models, where model 1 estimated the impact of baseline BMI status on expenditures and model 2 shows the additional effect of BMI change over the last 10 years. In each of the two models, the odds ratio of drug use was modelled in the first step and the amount of total drug expenditures in the second step.

**Table 4:** Impact of baseline BMI and BMI change on drug expenditures – two-step regression analysis

	**Model 1**				**Model 2**			
	**Impact of baseline BMI only**				**Impact of baseline BMI and BMI change**			
	**1. Probability**		**2. Amount of costs if >0**		**1. Probability**		**2. Amount of costs if >0**	
	**n = 2,946**		**n = 2,003**		**n = 2,946**		**n = 2,003**	
**Parameter**	**Odds ratio**	**p-value**	** Exp(est)**^a^	** p-value**	**Odds ratio**	**p-value**	** Exp(est)**^a^	** p-value**
**Intercept**	0.164	0.055	37.462	<.0001	0.168	0.058	33.191	<.0001
**Age**	0.974	0.449	0.951	0.018	0.971	0.399	0.950	0.008
**Age squared**	1.001	0.010	1.001	0.004	1.001	0.007	1.001	0.001
**Sex: female**	2.179	<.0001	0.967	0.582	2.162	<.0001	0.960	0.462
**SES**^**b,d**^		0.347		0.112		0.324		0.045
**Lower middle class**	1.036	0.817	0.922	0.389	1.034	0.830	0.929	0.387
**Middle middle class**	0.984	0.913	0.894	0.256	0.987	0.930	0.914	0.314
**Upper middle class**	1.247	0.137	1.084	0.400	1.253	0.129	1.095	0.292
**Upper class**	1.108	0.514	1.079	0.476	1.120	0.472	1.123	0.233
**BMI**^**c,d**^		<.0001		0.001		<.0001		<0.0001
**Overweight**	1.188	0.087	1.132	0.089	1.189	0.086	1.126	0.073
**Moderately obese**	1.776	<.001	1.431	<.001	1.769	<.001	1.426	<.0001
**Severely obese**	4.068	<.0001	1.361	0.025	4.134	<.0001	1.417	0.005
**BMI change**	–	–	–	–	1.037	0.087	1.055	<.0001
**Scale**	2.718	–	1.839	–	2.718	–	2.099	–
**AIC**	3228		14385		3227		14322	

^a^Exponents of regression estimates are reported, that can be interpreted as factors.

Reference: ^b^Lower social class; ^c^Normal weight.

^d^p-values for overall impact of group variable are given.

According to model 1, compared with the normal weight group, the odds of pharmaceutical intake were about 78% higher for moderately obese and more than fourfold for severely obese participants (both significant). Regarding the participants with pharmaceutical intake, expenditures were about 40% higher for moderately and severely obese participants. Regarding the possible confounders, age showed a significant U-shaped effect on the amount of total expenditures. Women had significantly higher odds of causing expenditures than men. However, there were no significant differences between men and women regarding the amount of expenditures. The overall impact of SES was not significant in this model.

In an extended model, we tested the additional impact of weight change over the last 10 years. The respective results are also shown in Table 2 (model 2): BMI change has no significant effect on the odds of drug intake (p = 0.087), but (for those with positive expenditures) does have an effect on the amount of drug expenditures. Thus, on average, a one-point BMI gain over the last 10 years is associated with 5.5% higher expenditures compared with someone with a constant BMI, among those that have positive expenditures. Compared with model 1, however, additionally adjusting for BMI change hardly affected the regression results for baseline BMI or for the other variables. Only the overall effect of SES becomes significant in the second step of this extended model.

**Figure 1:**
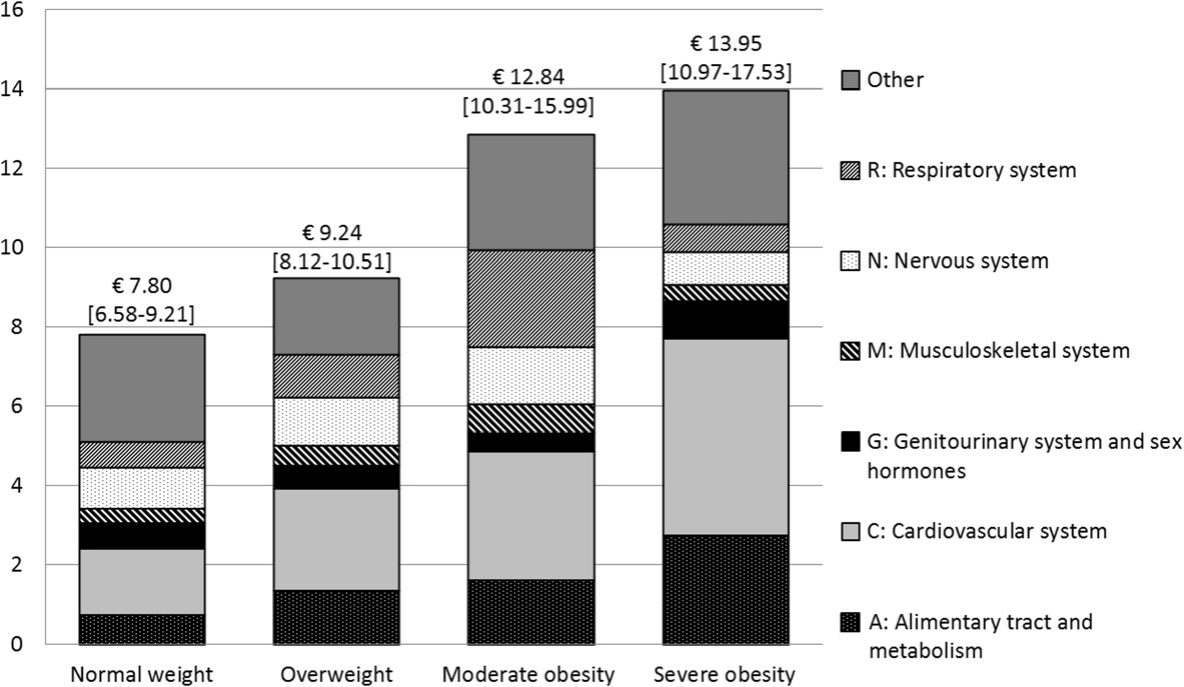
**Adjusted mean expenditures per week (€) by ATC group and baseline BMI group [95% CI]**^**a**^**.**

Figure 1 shows the contributions of the six economically most important individual ATC groups to total adjusted expenditures for each BMI category. An increase in mean drug expenditures is especially noticeable for those pharmaceutical groups targeting the alimentary tract and metabolism (differences to normal weight significant for overweight (p = 0.002), moderate and severe obesity (p < 0.0001)) and the cardiovascular system (differences to normal weight significant for overweight, moderate and severe obesity (p < 0.0001)). Also compared to the normal weight group, expenditures for pharmaceuticals targeting the respiratory system are significantly higher in the moderately obese group (p = 0.006), but not for the severely obese (p = 0.790). Also, the composition of drug expenditure by ATC groups differed significantly across BMI groups. The ‘other’ group contains ATC groups for medicines targeting blood and blood-forming organs (B), dermatologicals (D), systemic hormonal preparations, excluding sex hormones and insulins (H), antiinfectives for systemic use (J), antineoplastic and immunomodulating agents (L), antiparasitic products, insecticides and repellents (P) and sensory organs (S).

## Discussion

The present study aims to analyse the impact of obesity and change in BMI on future pharmaceutical utilisation and expenditures based on data from two population-based health surveys in Germany.

The percentage of participants with future drug intake as well as the proportion of prescription drugs increases significantly with rising baseline BMI group. Also, after adjusting for age, sex and SES, moderately and severely obese people have significantly higher odds of future drug utilisation as well as higher future expenditures compared with normal weight participants. This is consistent with results from the existing international literature on the impact of BMI on pharmaceutical expenditures based on cross-sectional studies, e.g. [9-13]. BMI change has no significant additional effect on the odds of drug intake, but does have an effect on the amount of expenditures. On average, a one-point BMI gain over the last 10 years is associated with 5.5% higher expenditures compared with someone with a constant BMI. In total, 11% of the formerly overweight or obese participants succeeded in losing weight and changing to one of the lower BMI categories. Among those who have drug expenditures, on average, a one-point BMI decrease during the last 10 years was associated with 5% lower drug expenditures – independent from baseline BMI. The independent effect of weight change on costs is an important finding, which is confirmed by an additional analysis adjusting for current BMI instead of baseline BMI (in this case costs changed by almost 3% per BMI point gained/lost). This shows that successful prevention programmes, but also intervention programmes for already obese people, have a strong potential to reduce pharmaceutical costs in the long run.

The reported results show that an increase in mean drug expenditures is especially noticeable for those pharmaceutical groups targeting the alimentary tract and metabolism (A) and the cardiovascular system (C). In our sample, these groups particularly include drugs for diabetes and coronary heart disease. This is in line with the findings of an earlier study in the U.S [19] and seems quite plausible, as obesity is a known risk factor for diseases such as diabetes, dyslipidaemia, coronary heart disease and cardiac insufficiency [14].

This is the first study analysing the longer term effects of BMI and the effect of BMI change on pharmaceutical utilisation and expenditures for Germany in a bottom-up approach. This is possible because of the longitudinal design of the MONICA/KORA cohort study, which provides patient-level information on drug intake (including out-of-pocket expenditures), measured BMI and sociodemographic variables. Another advantage of this approach is the possibility of comparing expenditures in population subgroups, for example with respect to sociodemographic variables and BMI. Although analyses based on comprehensive administrative statistics might give better estimates of the actual level of expenditures for the respective institution (e.g., health insurance), they mostly do not include patients’ out-of-pocket-expenditures. Furthermore, these studies are often limited to cases of diagnosed obesity rather than measured weight for height and do not allow distinction between different degrees of obesity.

However, the study has some limitations. First, the MONICA/KORA survey is not fully representative of the population in Germany; for example, the two health surveys are limited to people of German nationality [23], and earlier studies have shown that obesity is more prevalent in migrant cohorts [28]. Another reason for the uncertainty of the results is drop-out. Regarding the study sample for this analysis, a total of 1,877 people dropped out from the Baseline survey S3 (1994/95) to the Follow-up F3 (2004/05): of these, 19.6% were moderately obese at baseline and 7.3% were severely obese. Compared with the weight distribution in the analysed sample, more severely and moderately obese people dropped out than normal weight and overweight people. Therefore, it cannot be excluded that people with a high utilisation of health care services, for example owing to greater age or illness, have dropped out before the Follow-up KORA survey was conducted. Therefore, future pharmaceutical expenditures due to obesity class (change) may be underestimated in this analysis, particularly in the case of moderately and severely obese participants.

Moreover, the statistically insignificant results of severely obese participants and users might result from low statistical power owing to the relatively small number of cases in this group compared with the other weight categories.

Although the problem of recall error should be small considering the short time period, it cannot be excluded as participants are asked to provide information retrospectively – in this case, to state the utilisation of pharmaceuticals for the previous 7 days.

The estimation of drug expenditures was based on several assumptions that may have caused under- or overestimation. To reduce the uncertainty of drug utilisation, sensitivity analyses were conducted. First, if pharmaceuticals were not taken regularly, we assumed one intake per week as a conservative base analysis. An alternative assumption of daily intake leads to an increase in mean expenditures of around 29%. Furthermore, sensitivity analysis was performed regarding legal price discounts. Al-though all these univariate changes affect the extent of expenditures in total, they barely affected the differences between BMI classes. Discount contracts between the pharmaceutical industry and health care insurers, as introduced in 2003, could not be taken into account as this information is not publicly available.

In this study, we estimated the expenditures for drug consumption based on utilisation during 1 week. The actual expenditures might be even higher if packages are only partly used and leftovers are thrown away. Moreover, as utilisation of pharmaceuticals was requested for the last 7 days, the extrapolated yearly expenditure estimates should be interpreted with caution.

SES was included as a confounder in statistical analyses because it may influence health care utilisation as an ‘enabling factor’ [36]. Yet, it has to be noted that SES may also be associated with overweight and obesity, but causality is not trivial [37,38]. Although low income might have a negative impact on health behaviour resulting in weight gain, overweight and obesity could also impede labour market outcomes and cause lower wages [5]. However, a recalculation of the regression model without SES as a confounding variable did not change our results.

## Conclusions

The results of this analysis suggest that obesity is a strong predictor of future drug utilisation and associated expenditures in adults, and therefore highlight the necessity of timely and medically effective intervention and prevention programmes. Moreover, BMI change is shown to be an independent predictor of drug expenditures. This may be an important finding regarding the implementation and evaluation of obesity prevention programmes: next to BMI reduction in overweight and obese adults, the sustainable prevention of BMI gain should be seen as an important goal and an indicator for the success of an intervention. Based on these findings, medically effective obesity prevention and intervention programmes have considerable potential to reduce short- and long-term drug expenditures.

Yet, the relationship between BMI and health care expenditures cannot be clearly defined as long as a complex analysis including all causal relations between obesity and chronic diseases is still lacking. Higher health care utilisation might also be caused by a non-observable disease. Owing to the complexity of obesity as a health problem, the excess-cost approach was chosen for this analysis, assuming that all excess utilisation is related to excess weight [39]. In order to completely understand the interaction between weight status, weight development and the associated future health care utilisation and costs from a lifetime perspective, further research is necessary. Besides longer-term cohort- and modelling studies, this should also include methodological research on cost assessment and measurement as well as the economic evaluation of effective obesity prevention programmes.

## Competing interests

The authors declare that they have no competing interest.

## Authors’ contributions

CMT, PM and SBW developed the design and analysis plan of this study. CMT and PM performed statistical analyses. CMT drafted the manuscript. RH, JJ and MH were involved in the coordination/conception of the KORA-study. All authors contributed to interpretation of findings, critically reviewed each draft of the manuscript, contributed to writing and approved the final manuscript.

## Pre-publication history

The pre-publication history for this paper can be accessed here:

http://www.biomedcentral.com/1472-6963/13/424/prepub
